# Observation of prior light emission before arcing development in a low-temperature plasma with multiple snapshot analysis

**DOI:** 10.1038/s41598-022-25550-2

**Published:** 2022-12-05

**Authors:** Si-jun Kim, Young-seok Lee, Chul-hee Cho, Min-su Choi, In-ho Seong, Jang-jae Lee, Dae-woong Kim, Shin-jae You

**Affiliations:** 1 Applied Physics Lab for PLasma Engineering (APPLE), Department of Physics, Chungnam National University, Daejeon, 34134 Republic of Korea; 2 Samsung Electronics, Gyeonggi-do, 18448 Republic of Korea; 3 Department of Plasma Engineering, Korea Institute of Machinery and Materials (KIMM), Daejeon, 34104 Republic of Korea; 4 Institute of Quantum Systems (IQS), Chungnam National University, Daejeon, 34134 Republic of Korea

**Keywords:** Applied physics, Plasma physics

## Abstract

Arcing is a ubiquitous phenomenon and a crucial issue in high-voltage applied systems, especially low-temperature plasma (LTP) engineering. Although arcing in LTPs has attracted interest due to the severe damage it can cause, its underlying mechanism has yet to be fully understood. To elucidate the arcing mechanism, this study investigated various signals conventionally used to analyze arcing such as light emission, arcing current and voltage, and background plasma potential. As a result, we found that light emission occurs as early as 0.56 μs before arcing current initiation, which is a significant indicator of the explosive development of arcing as well as other signals. We introduce an arcing inducing probe (AIP) designed to localize arcing on the tip edge along with multiple snapshot analysis since arcing occurs randomly in space and time. Analysis reveals that the prior light emission consists of sheath and tip glows from the whole AIP sheath and the AIP tip edge, respectively. Formation mechanisms of these emissions based on multiple snapshot image analysis are discussed. This light emission before arcing current initiation provides a significant clue to understanding the arcing formation mechanism and represents a new indicator for forecasting arcing in LTPs.

## Introduction

Arcing, also called sparking or flashover, is known as a transient discharge and is a ubiquitous phenomenon in high-voltage applied systems ranging from ultra-high vacuum^1–4^ to atmospheric pressure^5,6^. Since arcing itself is a high-density and high-temperature plasma that causes severe damage to material surfaces^7–12^, it has received enormous attention in research fields as well as industry for over 120 years^13^. Numerous studies have revealed arcing *initiation* mechanisms in direct current (DC) and radio frequency (RF) voltage environments in ultra-high vacuum, like field emission^14^ and thermo-field electron emission^15^ induced by a high electric field, which is on the order of several tens of GV/m and realized under circumstances of several tens of kV with micro-gaps^16^. Besides ultra-high vacuum conditions, arcing has also been observed in middle vacuum with low-temperature plasmas (LTPs), and several studies have investigated its ignition mechanisms^17–20^. But considering its long history, the *evolution* mechanisms of arcing are still not well understood^13^. In a DC environment, local ohmic heating induced by a field emission current has been known as a significant process in arcing evolution as it provides neutrals to a vacuum space by thermal evaporation and ignites arcing, whereas in an RF environment, most experimental results have not been in agreement with the ohmic heating mechanism^21^. Recently, Norem et al.^13^ have introduced a unified RF arcing model and argue that Maxwell stress induced by a high electric field can statistically cause surface cracks and atomic asperities without local ohmic heating, which can provide neutrals to the vacuum space and produce field emission. Their model well explained experimental results, but as mentioned in their paper, further improvement of the model based on basic measurement data at other RF frequencies is still required.

With respect to arcing in LTPs, there is no universal model of arcing evolution, although several studies have investigated it in LTP environments. Anders et al.^17^ studied arcing on a powered metal target (powered electrode) in a plasma sputtering deposition process and contended that dielectric contaminants on a metal target are the seed of arcing. Here, a localized electric field is induced by a charging of plasma ions on the dielectric contaminants such as the surfaces of a thin dielectric film and dielectric inclusions. Such an electric field is high enough to create field emission, induce ohmic heating, and finally ignite arcing. It has also been reported that arcing can be ignited without dielectric contaminants on a metal surface. Yin et al.^22,23^ investigated the origin of arcing in a hollow cathode discharge system designed to increase the plasma potential for frequent arcing generation. They observed that arcing occurs only on grounded metal surfaces, such as the vacuum chamber wall and grounded electrode. To investigate the mechanism, by utilizing particle-in-cell simulation and a circuit model they found that their experimental configuration produces a non-vanishing ion sheath only on the grounded metal surfaces^18^, where the sheath refers to an ion space-charge region between a plasma and a material that provides an ion transport channel from the plasma to the material surface. Based on Yin’s qualitative analysis, ions from plasma continuously bombard a grounded metal surface through the non-vanishing sheath, and this causes field emission and/or secondary electron emission from the surface. Provided that these emissions are localized to a sharp and tiny spot, local ohmic heating is induced and arcing is initiated^24^. A similar observation reported by Boswell et al.^19^ supported Yin’s mechanism, where the temporal development of long-lived arcing was measured by a high-speed camera in a helicon plasma reactor system. They pointed out that the arcing showed a similar behavior as a cathodic arc, in line with Yin’s explanation.

On the other hand, there is also an experimental result from a similar experimental configuration as Yin’s that arcing occurs only on dielectric surfaces in LTPs. Kim et al.^20^ investigated arcing in a capacitively coupled plasma system with an intensified charge-coupled device (ICCD) camera. They coated a part of the electrode with an oil (dielectric) to investigate whether arcing occurs on the metal or the dielectric surface. The recorded ICCD camera images showed that arcing appeared not on the metal surface but on the oil-soiled surface. Based on their measurements, they introduced two possible mechanisms with rough estimation. The first is a breakdown in the sheath, called a Paschen breakdown: an increase in the local pressure in the sheath by plasma-enhanced evaporation of the oil surface induces an ionization avalanche in the sheath and resulting breakdown. The second mechanism is a dielectric breakdown of the oil: since the thickness of the oil coating is a few micrometers and the ion surface charging potential is a few tens of volts, the electric field (>10$^{7}$ V/m) is beyond the dielectric strength of the oil and a dielectric breakdown occurs.

These opposing observations and various speculations about the arcing formation mechanism in LTPs point to its highly complicated nature. Hence, studying arcing in the initiation phase in detail is required.

In the present work, we investigated arcing generated on an arcing inducing probe (AIP) under a capacitively coupled plasma environment with an ultra-high-speed camera. This paper is organized as follows. In the second section, details for the experimental setup are described, such as background plasma generation, arcing localization and enhancement, and multiple snapshot analysis method. Then, in the third section, recorded arcing images are presented and qualitative analysis is discussed. In the final section, we conclude this paper with a summary of the findings and discussions.

## Experimental setup

In this section, the background LTP system, arcing enhancement, and measurement system are described. A capacitively coupled plasma (CCP) source was used to maintain a background LTP, as shown in Fig. 2. Argon gas (99.999% purity) of 50 standard cubic centimeters per minute (sccm) flows into a vacuum vessel via a mass flow controller (MASS-FLO, MKS Instruments Inc.), and a vacuum pump (DS 302, Varian Inc.) draws the argon gas and maintains a vacuum vessel pressure of 166.4 mTorr, which is measured by a vacuum gauge (1 Torr Baratron gauge, MKS Instrument Inc.). A 13.56 MHz RF power (P_RF_) of 40 W produced by an RF generator (RFPP RF5S, Advanced Energy Industries, Inc.) is applied to a powered electrode through multiple components: a coaxial cable, an RF matcher (Path Finder, Plasmart Inc.) designed to deliver RF power without reflection, and an RF feeding line. As a result, a background LTP forms. As the background LTP expanding to the elbow-shaped pumping port causes frequent arcing ignition inside the pumping port, a mesh grid is installed at the entrance of the pumping port to prohibit the background LTP expansion.

**Figure 1:**
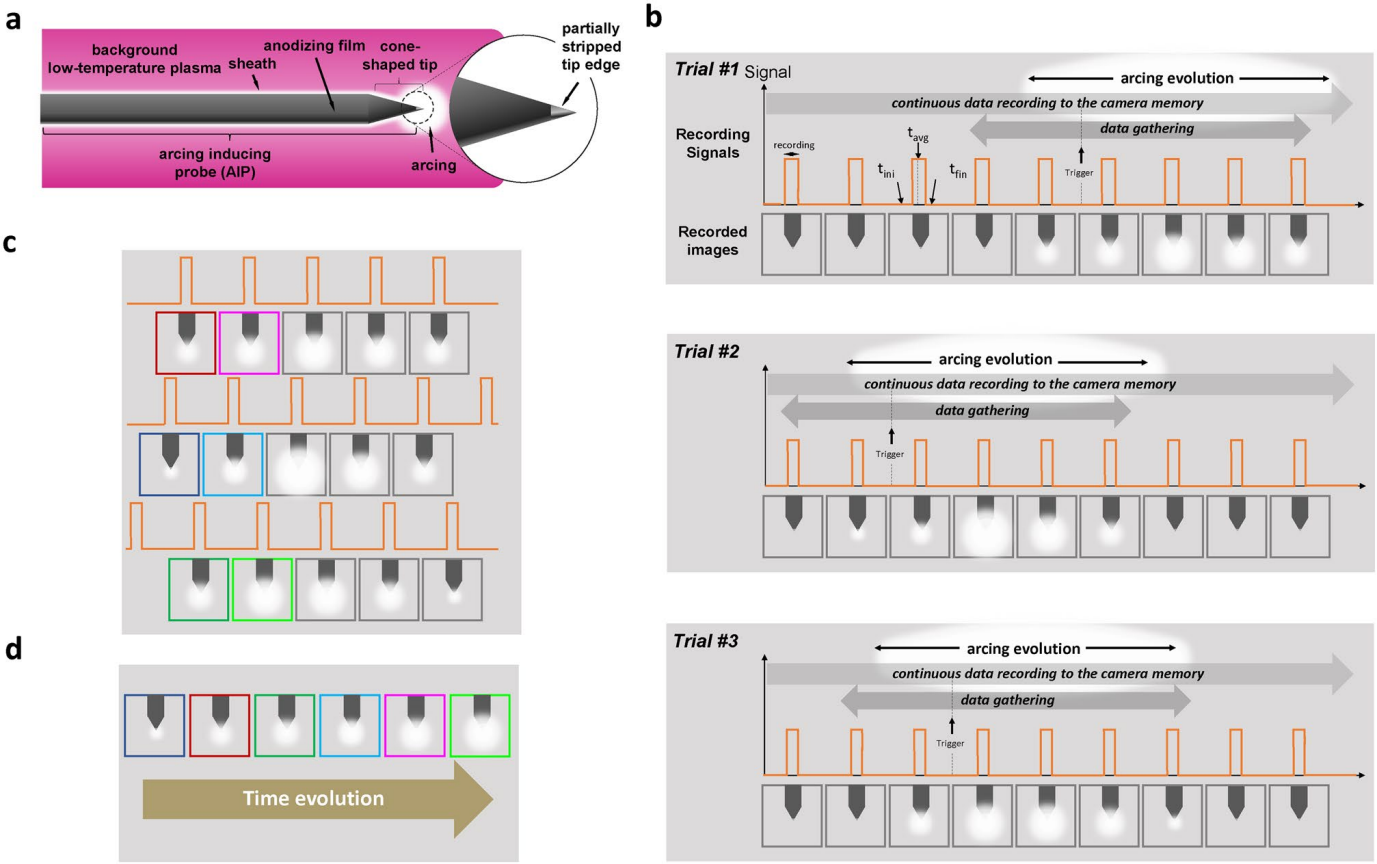
(**a**) Schematic diagram of the arcing inducing probe (AIP). (**b**) Schematic diagram of the multiple snapshot analysis method with several trials. The terms $t_{\textrm{ini}}$, $t_{\textrm{fin}}$, and $t_{\textrm{avg}}$ mean the beginning, termination, and average time of the ultra-high-speed camera recording, respectively. (**c**) Process of gathering arcing images and (**d**) sorting the images in time order.

As arcing occurs randomly in space, we inserted the AIP designed to localize arcing on the tip edge, as shown in Fig. 1a. The AIP is an aluminum rod covered with an anodizing film, and its tip edge has a partially stripped cone shape where arcing mostly arises, as shown in Fig. 1. Furthermore, as arcing occurs randomly in time, we continuously recorded the AIP tip edge with the ultra-high-speed camera connected to an arcing trigger system, recording images before and after the trigger moment for several trials as shown in Fig. 1b. Then, the recording images are gathered and sorted in time order as shown in Fig. 1c, d. With this setup, arcing in the initial phase can be analyzed despite its random characteristics.

In fact, in a typical CCP source for research purposes, arcing rarely arises as the RF power is low, typically ranging in several hundred watts. To increase the arcing rate in this power range, a negative bias by a DC power supply (APH1000m, KEPCO Inc.) is applied to the AIP, as shown in Fig. 3a. This increases the potential difference between the background LTP and the AIP tip edge ($\Delta V$), which enhances arcing ignition and arcing localization on the tip edge. Since the $\Delta V$ is larger than the difference between the plasma and the wall, arcing generation probability on the AIP tip is higher than that on either a vacuum chamber wall or other parts inside the chamber. In general, ions from the LTP strike the AIP tip with an energy similar to the $\Delta V$. The ion bombardment enhances field emission by increasing electron tunneling probability and transferring its kinetic energy to the emitting spot.

Furthermore, a $\Pi$-type RF matcher having no DC blocking capacitors is employed. This configuration allows DC current to flow through the electrode–background LTP–grounded electrode and causes an increase in plasma potential as much as the electrode voltage to suppress drastic electron loss on the grounded chamber wall^24^ and as a result, enhances the arcing rate^20,22^.

**Figure 2:**
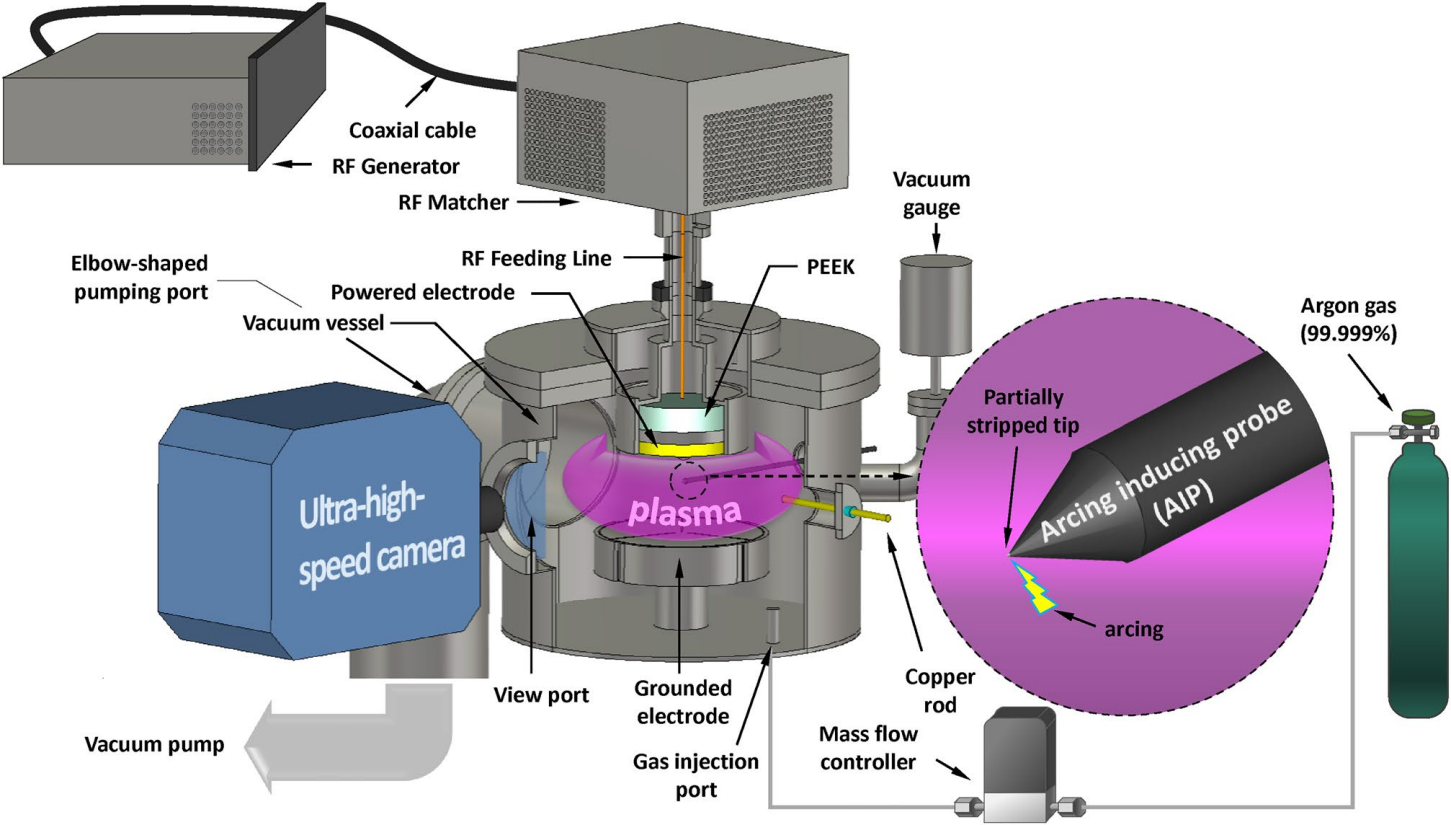
Semi-cross-sectional schematic of the experimental setup configuration.

**Figure 3:**
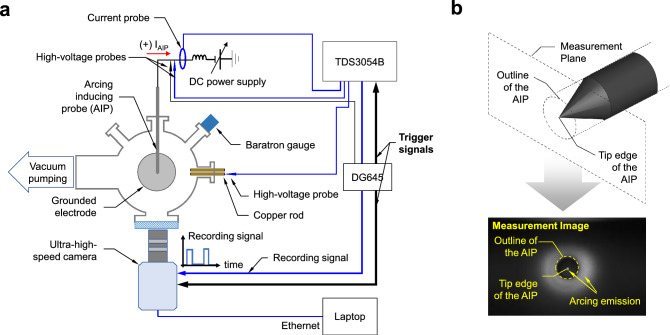
(**a**) Planar schematic of the experimental setup. (**b**) Measurement plane of the ultra-high-speed camera and example measurement image.

**Figure 4:**
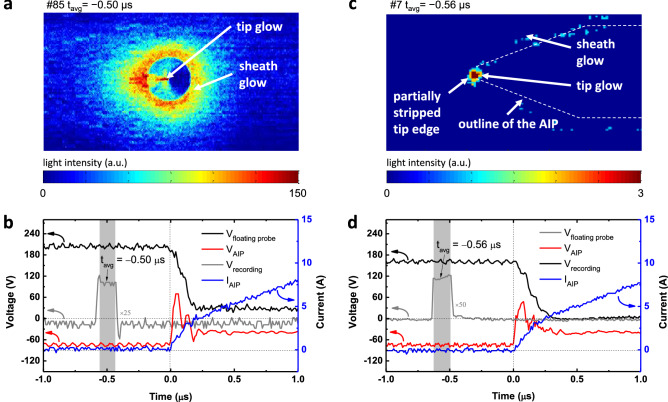
(**a, c**) Recorded images of the front view (**a**) and side view (**c**) with a $t_{\textrm{recording}}$ of 0.154 $\mu$s. (**b, d**) Floating probe voltage ($V_{\textrm{float}}$), voltage and current of the AIP ($V_{\textrm{AIP}}$ and $I_{\textrm{AIP}}$), and recording voltage signal of the ultra-high-speed camera ($V_{\textrm{recording}}$) over time for front-view (**b**) and side-view (**d**) measurement. The color bar denotes the light intensity. All measurements were taken under the following conditions: P$_{\textrm{RF}}$ of 40 W to maintain the background plasma, pressure of 166.4 mTorr, and argon injection of 50 sccm.

**Figure 5:**
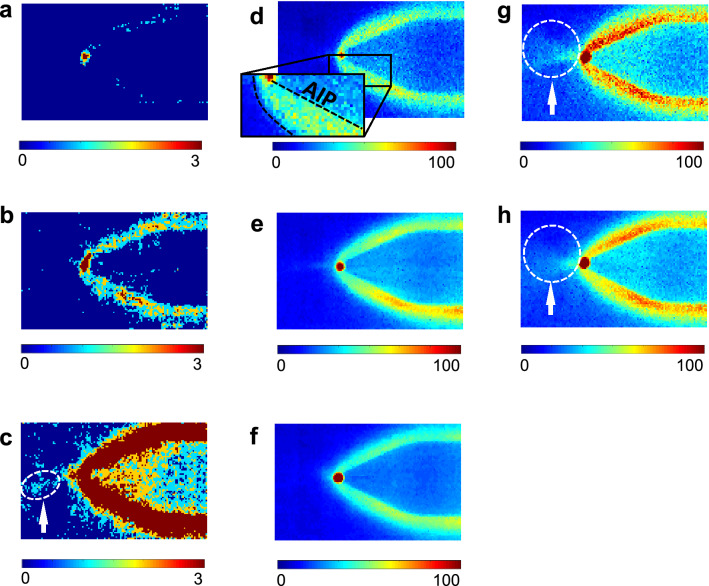
Recorded arcing evolution side-view images at *t*$_{\textrm{avg}}$ of (**a**) −0.56 $\mu$s, (**b**) −0.53 $\mu$s, (**c**) −0.44 $\mu$s, (**d**) −0.04 $\mu$s, (**e**) (**b**) 0.54 $\mu$s, (**f**) 0.71 $\mu$s, (**g**) 0.12 $\mu$s, and (**h**) 0.38 $\mu$s with a $V_{\textrm{AIP}}$ of $-75$ V, P$_{\textrm{RF}}$ of 34 W, the pressure of 147.3 mTorr, and argon injection of 20.0 sccm.

**Figure 6:**
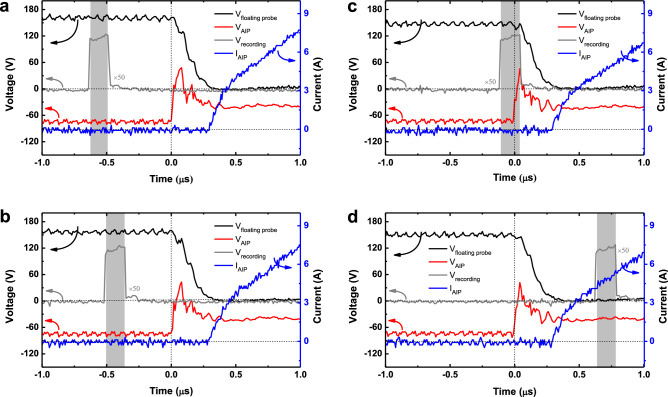
Floating probe voltage ($V_{\textrm{float}}$), voltage and current of the AIP ($V_{\textrm{AIP}}$ and $I_{\textrm{AIP}}$), and recording voltage signal of the ultra-high-speed camera ($V_{\textrm{recording}}$) at a $V_{\textrm{AIP}}$ of $-75$ V, P$_{\textrm{RF}}$ of 34 W, the pressure of 147.3 mTorr, and argon injection of 20.0 sccm. The recording times, $t_{\textrm{avg}}$, are (**a**) −0.56 $\mu$s, (**b**) −0.44 $\mu$s, (**c**) −0.04 $\mu$s, and (**d**) 0.71 $\mu$s. Each images of Fig. 6(a)–(d) recorded at the same time of Fig. 5(a)–(e), respectively.

**Figure 7:**
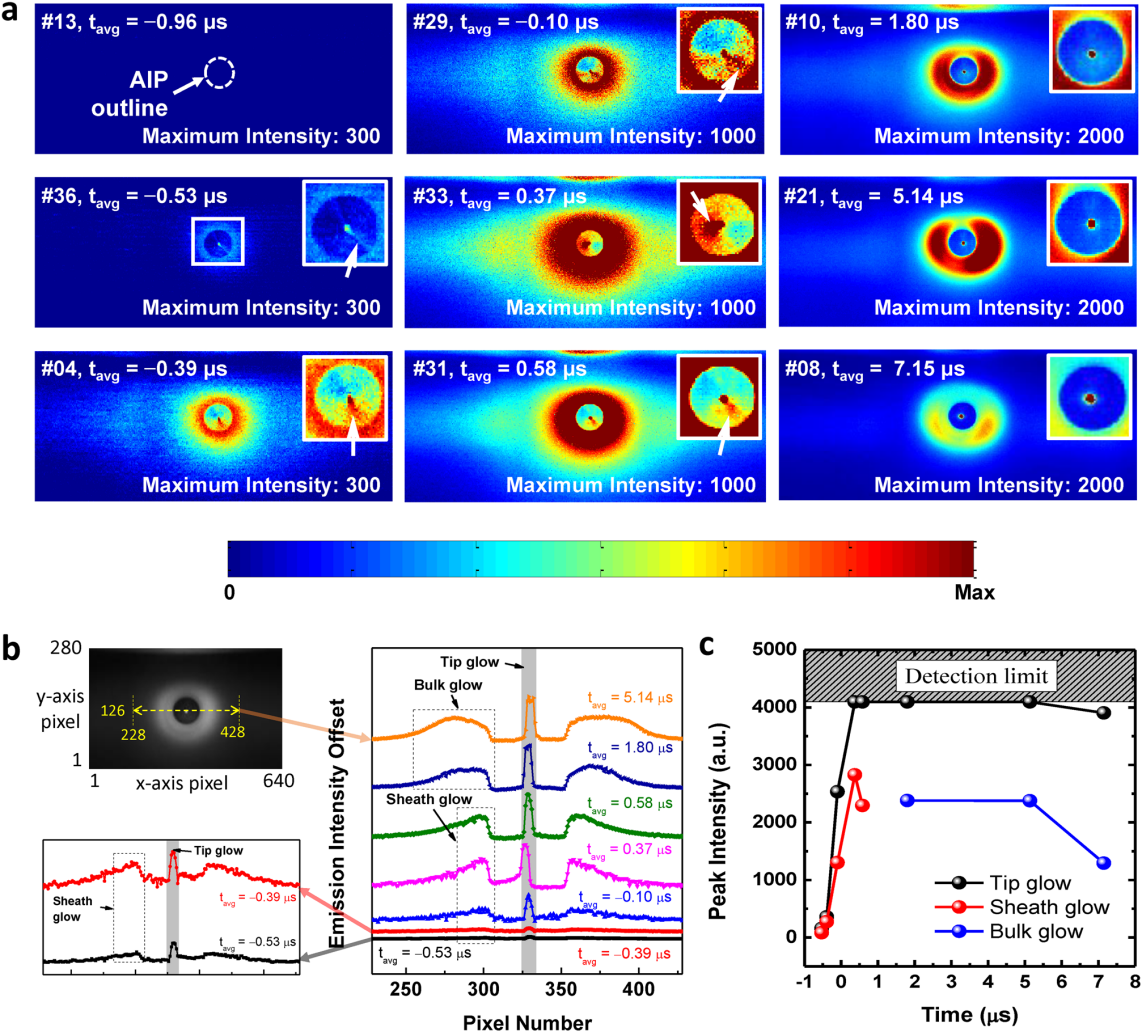
(**a**) Recorded images from a front-view measurement at a $V_{\textrm{AIP}}$ of $-75$ V, P$_{\textrm{RF}}$ of 40 W, pressure of 166.4 mTorr, and argon injection of 50 sccm. (**b**) Intensity of the images in (**a**) at the y-axis pixel value of 126 and x-axis pixel values from 228 to 428. (**c**) Peak intensity of each glow type over time.

To measure the light emission from arcing, an ultra-high-speed camera (Fastcam SA-Z, Photron) designed to measure visible light with a monochrome sensor is set up in front of the view port to image the AIP, as shown in Figs. 2 and 3a. Figure 3b shows a schematic diagram of the measurement plane focused on the tip edge of the AIP and a measurement image marking the outline of the AIP body and tip edge. For all measurement data in this study, the shutter open period of the camera is fixed at the shortest time, $0.154~\mu$s. Detailed image analysis is discussed in the next section. Besides the arcing light emission measurements, to measure the electrical characteristics of arcing, a high-voltage probe (P5100A, Tektronix Inc.) and current probe (TCPA300, Tektronix Inc.) are connected with an oscilloscope (TDS3054B, Textronix Inc.), as shown in Fig. 3a. Then to measure the plasma potential, a copper rod, called a floating probe, is immersed into the plasma and the high-voltage probe connected to the oscilloscope measures its voltage; this voltage well follows the plasma potential^25^, as proved in Fig. S1 (Supplementary Information). The triggering of an arcing event is described in the Methods section.

As arcing occurs randomly in space inside a vacuum chamber, direct measurements of arcing signals such as voltage and current are impractical. Indirect methods have been adapted, such as measuring the voltage of the floating probe^19,20,24^, RF voltage/current of the powered electrode, emission spectrum of plasma^26^, and acoustic emission^27^. In this experiment setup, the AIP and additional bias were introduced to localized arcing. The agreement of artificial arcing generation with natural arcing can be verified by comparing it with electrical signals as follows. After arcing development, abrupt voltage drops of both a floating probe^19,24^ and a powered electrode^26^ have been reported. In this experiment setup, the arcing generated on the AIP tip edge shows a good agreement with those properties, as shown in Fig. S1a and b (supplementary information).

## Results and discussion

### Observation of light emission before arcing current initiation

This section focuses on the *observation* of light emission prior to arcing current initiation, with further analysis following in the next section. Figure 4a shows a recorded image from a front-view arcing measurement and Fig. 4b shows the corresponding waveforms of the voltages of the floating probe ($V_{\mathrm{floating~probe}}$), the AIP ($V_{\textrm{AIP}}$), and the recording ($V_{\textrm{recording}}$), as well as the current of the AIP ($I_{\textrm{AIP}}$). Here, the zero time is treated as the moment of arcing current initiation. As shown in the recorded image and $V_{\textrm{recording}}$, light emission at a $t_{\textrm{avg}}$ of $-0.50~\mu$s is clearly observed on both the surface and tip edge of the AIP, named sheath and tip glows, respectively. The sheath glow intensity shows the highest value on the AIP outline (surface), whereas the tip glow intensity is highest on the AIP tip edge. At this moment, $V_{\mathrm{floating~probe}}$, $V_{\textrm{AIP}}$, and $I_{\textrm{AIP}}$ are stable compared to after arcing current initiation when all signals drastically change, as shown in Fig. 4b. This result means that the light emission before arcing current initiation has no influence on the background LTP. To further investigate this light emission, the initiation time of the emission is estimated. As previously mentioned, since arcing occurs randomly in time, we amassed a large number of experimental trials, sorted them, and figured out the earliest initiation time. Here, we assume that the arcing occurring in different trials is similar, as proved in next paragraph. Among all trials, the earliest initiation time was revealed as $-0.56~\mu$s in a side-view experimental configuration, where the positions of the floating probe and the AIP were exchanged. Prior to this initiation time, no light was observed. A recorded image and the waveforms of this trial are shown in Fig. 4c, d, respectively. Clear tip glow and indistinct sheath glow are observed. Hence, based on the measurement, light is emitted as early as $0.56~\mu$s before arcing current initiation while the background LTP is in a stable state.

In several vacuum arc studies with high-temporal resolution analysis^1,3,28^, this prior light emission was not reported. As the observation of light emission before arcing current initiation is an unprecedented finding, we discuss here the measurement validity in terms of the following: (i) reproducibility and consistency between inter-experimental data, and (ii) time delay of the instruments. There are two clear pieces of evidence for the validity of our measurements. First, the light emission measured at similar evolution moments in different experimental trials exhibits similar emission patterns, as shown in Fig. 4a, c, as well as other in measurements shown in Fig. S2 (Supplementary Information). Furthermore, the evolution behavior shows a similar trend when comparing Fig. 5a–f, which shows recorded images from side-view measurements in order of time called multiple snapshot image analysis, with Fig. S2b (Supplementary Information). Second, the time for the explosive increase in the emission intensity exactly corresponds to the time of the $I_{\textrm{AIP}}$ increase, as shown in Fig. 6. In the early phase from 5a, b, the emission intensity gradually rises on the order of several tens, whereas it explosively grows after $I_{\textrm{AIP}}$ increases from 5d–f (see the color bar scale), on the order of hundreds.

To examine the instrumental time delay, we consider the time delays in (i) the coaxial cable delivering the trigger signal from the DG645 to the oscilloscope and the ultra-high-speed camera, as shown in Fig. 3, and (ii) the high-voltage probe cable. Firstly, the time delay induced by the coaxial cable was about 0.11 $\mu$s; for the raw data from the oscilloscope, the time difference between the trigger moment and arcing initiation is about 0.11 $\mu$s, as shown in Fig. S3 (Supplementary Information). Furthermore, since the same length of coaxial cable was used for the ultra-high-speed camera, there is no trigger time delay between the oscilloscope and high-speed camera. The trigger signal, however, is related not to the *recording* but to the *gathering*. Specifically, data was continuously recorded in the instrument memory, while the trigger ordered the gathering of the data and its transport to computer memory. Secondly, the time delay (or propagation delay) in a high-voltage probe cable with a length of 2.0 m is about 6.1 ns as provided from the manufacturer^29^, which is negligible compared to 0.63 $\mu$s.

### Emission characteristics and formation mechanism based on multiple snapshot image analysis

Although the exact mechanisms of the prior light emission as well as of arcing itself in LTPs have yet to be fully understood, we describe here the emission characteristics and explain the formation mechanism with rough estimation. Based on multiple snapshot analysis on a large time scale, we found that the light emission can be classified into three types by evolution in time: tip, sheath, and bulk glow. The prior light includes the tip and sheath glows, while the bulk glow evolves after $I_{\textrm{AIP}}$ initiation. In the following subsections, we discuss their characteristics in order of evolution.

#### Tip glow

The tip glow is characterized as light highly localized on a tiny spot where arcing arises, as distinctly shown in Figs. 4a and 5. This glow type presents a spot-like shape wherever arcing arises, on either the probe tip edge or the AIP body. As seen in both side- (Fig. 5) and front-view (Fig. 7a) measurements, the tip glow has a circular shape, meaning it is spherical and localized on the cone-shaped tip edge during the whole arcing process from beginning to end. Furthermore, as all recorded images show, the tip glow produces the highest emission intensity compared to the other glow types as in Fig. 7b; therefore, most energy is consumed on this spot and explosive damage may result from tip glow. Additionally, based on Fig. S4 (Supplementary Information), the tip glow intensity is several thousand times higher than the background LTP.

As tip glow is the primary process during arcing evolution, we mainly focus on explaining the formation mechanism of this glow type. Speculations on the formation mechanism of tip glow are briefly discussed, with experimental evidence and estimations presented as follows. In the first phase, ion surface charging on the anodizing film of the AIP creates a surface potential as large as the plasma potential, and a high electric field forms between the film surface and negatively biased asperities, which are sharp pinnacles on the atomic scale^13^. Then ion-assisted field emission and resulting local ohmic heating on the asperities produce field-emitted electrons, called ectons, and evaporated metal atoms. These ectons are accelerated by the electric field in the non-collapsing ion sheath and collide with background argon atoms and evaporated metal atoms. Inelastic collisions such as ionization and excitation produce primary electrons and photons, respectively. These primary electrons also collide with the above atoms and finally induce an electron avalanche. Here, it is noted that as evaporated metal atoms have a lower threshold energy for collisions than that of argon, their mean free path is much shorter than that of argon. This leads to abundant collisions in the sheath. Meanwhile, in general, at low pressure, such primary electrons or secondary electrons rarely collide with the background gas in the sheath as their mean free path is much longer than the sheath thickness, and thus they barely play a role in collisions. During the avalanche process, atomic excitation also intensifies and high-density photons from the de-excitation of excited atoms are released. Through this process, high-density plasma is generated, and high-intensity emission is released. In this case, the recorded images measure the direct emission light of excited species since the time scales of the excitation ($\tau _{\textrm{ex}}$) and de-excitation ($\tau _{\textrm{rad}}$) are a few nanoseconds. Time $\tau _{\textrm{ex}}$ is roughly estimated by $\tau _{\textrm{ex}}\sim n_{\textrm{Al}}\sigma _{\textrm{ex,Al}}\bar{\textrm{v}}$, where $n_{\textrm{Al}}$ is the atomic density of aluminum, $\sigma _{\textrm{ex,Al}}$ is the electron impact excitation cross section of aluminum, and $\bar{\textrm{v}}$ is the average relative velocity ($|\bar{\textrm{v}}|$) between an aluminum atom and electron ($|\bar{\textrm{v}}|\equiv |\bar{\textrm{v}}_{\textrm{e}}-\bar{\textrm{v}}_{\textrm{Al}}|\approx \bar{\textrm{v}}_{\textrm{e}}$ with $\bar{\textrm{v}}_{\textrm{e}}$ and $\bar{\textrm{v}}_{\textrm{Al}}$ the average electron and aluminum atom velocities, respectively). Assuming that $n_{\textrm{Al}}$ is $10^{\textrm{15}}$ cm$^{-3}$ for a minimum vapor pressure equaling the background argon pressure, $\sigma _{\textrm{ex,Al}}$^30^ is $10^{-\textrm{15}}$ cm$^{2}$ and $\bar{\textrm{v}}$ is $10^{8}$ cm/s for an electron temperature of 3 eV. Furthermore, provided that a photon is released by de-excitation through dipole radiation, time $\tau _{\textrm{rad}}$ is defined^31^ as $\frac{12\pi \epsilon _{0}\hbar c^{3}}{\omega ^{3}p_{\textrm{d0}}^{2}}$, where $\epsilon _{0}$ is the permittivity in vacuum, $\hbar$ is Planck’s constant, *c* is the speed of light, $\omega$ is the angular frequency of the photon ($\omega =e\varepsilon /\hbar$), $\varepsilon$ is the energy difference between excited and de-excited states, and $p_{\textrm{d0}}$ is the magnitude of the dipole moment ($ea_{\textrm{B}}$). For simplicity, we assume that $\varepsilon$ is the ionization energy and $p_{\textrm{d0}}$ is $ea_{B}$, where *e* is the elementary charge and $a_{\textrm{B}}$ is the Bohr radius of an aluminum atom.

In general, the arcing initiation mechanism in LTPs has been speculated to be field emission^18,20^ from asperities formed by a stochastic process with an intense electric field^32^. Based on our estimation, though, we found that ion bombardment has to be deemed to initiate field emission, in a process called ion-assisted field emission. The field emission current density ($J_{\textrm{FE}}$) is described with the Fowler–Nordheim equation with a local electric field ($E_{\textrm{local}}$)^33^, $J_{\textrm{FE}}=\frac{\textrm{A}(\beta E_{\textrm{local}})^{2}}{\phi }\exp {(-\frac{\textrm{B}\phi ^{3/2}}{\beta E_{\textrm{local}}}})$, where A and B are constants of $1.54\times 10^{-6}$ $\textrm{AeVV}^{-2}$ and 6.83 eV$^{-3/2}$ GV/m, respectively, $\phi$ is the work function which is 4.2 eV for aluminum metal^34^, and $\beta$ is a field enhancement factor that depends on the geometry of the field emission region and is usually given as 30–60 for copper^14^. The calculated threshold electric field for initiating field emission is 58.8 GV/m. Assuming $\beta$ as 100, the local electric field must be around 580 MV/m or hgiher. In the current experimental condition, however, the electric field is on the order of 10 MV/m, which is lower than the threshold; the voltage difference between the anodizing surface and the aluminum body is a few hundred volts (the film surface voltage comparable to the plasma potential in Fig. S1a of the Supplementary Information is about 200 V and the AIP bias voltage is $-75$ V), and the anodizing thickness is a few tens of micrometers. Herein, the ion bombardment effect lowers the field emission threshold by as much as 30 times since the image charge induced by ions thins the potential barrier width and increases the electron tunneling probability^35^. Then the threshold of ion-assisted field emission becomes a few GV/m, which is much lower than the calculated threshold without ion bombardment, 58.8 GV/m. In this way, an electric field on the order of several 10 MV/m with an enhancement factor of 100 is sufficient for field emission initiation.

After field emission by the local electric field ($\textbf{E}_{\textrm{local}}$), local ohmic heating on atomic-scale asperities and resultant metal evaporation occurs. Despite the small amount of current, an extremely large current density ($\textbf{J}_{\textrm{FE}}$) is created and high ohmic heating ($\textbf{J}_{\textrm{FE}}\cdot \textbf{E}_{\textrm{local}}$) is induced. As marked with white arrows in Fig. 5c, g, and h, an emission spreading out in front of the tip glow was observed, which indicates electron impact collisions between evaporated aluminum atoms and ectons; it has previously been shown that thermally evaporated atoms and field-emitted electrons show a spreading pattern from the source^14,36^. Furthermore, aluminum atoms have a lower threshold energy for excitation collisions as well as for ionization, which are on the order of a few electronvolts^37^. Since the average kinetic energy of the ectons ejected from the emitting area is a few electronvolts^38^ and they are accelerated by the electric field in the sheath up to several electronvolts, inelastic collisions are dominant in the non-collapsing ion sheath, ultimately producing a Townsend breakdown. We note that Yang et al.^39^ investigated copper vapor plasma via simulation and reported minimum breakdown voltages of 106–122 V at $5\times 10^{15}~\textrm{cm}^{-2}$. Considering that (i) the electron-impact ionization cross section of aluminum is ten times larger than those of copper and argon^40,41^, and (ii) the aluminum density is assumed as $10^{15}~\textrm{cm}^{-3}$ (few hundred millitorr of vapor pressure), breakdown is initiated within a sub-millimeter region in the non-collapsing sheath where the voltage difference between the background plasma and AIP tip is a few hundred volts. Finally, a high-density plasma forms, and the tip glow intensity and ecton current ($I_{\textrm{AIP}}$) simultaneously and explosively increase. As shown in Fig. 7c, which exhibits the peak intensity of each glow type over time, the tip glow intensity gradually increases prior to the $I_{\textrm{AIP}}$ increase, whereas after the $I_{\textrm{AIP}}$ increase it explosively increases, exceeding the detection limit of the ultra-high-speed camera. As shown in Fig. 7b, $I_{\textrm{AIP}}$ plays a role in the enhancement of the tip glow and does not change the mechanism in the early phase. The tip glow lasts up to several tens of microseconds.

#### Sheath glow

While the exact origin of the sheath glow is not clearly understood compared to that of the tip glow, we discuss here a possible formation mechanism. The sheath glow phenomenon occurs almost simultaneously with tip glow. At $-0.56~\mu$s in Fig. 5a, tip glow emerges while sheath glow begins to evolve, and by $-0.53~\mu$s in Fig. 5b, both center and sheath glows are apparent. The sheath glow is the brightest on the AIP surface and is released from the entire AIP sheath. As shown in Fig. 5d, the emission intensity increases closer to the tip edge, around which its shape seems not to resolve the entire cone shape but rather to round and combine with the tip glow. Together with the result that the tip glow evolves earlier than the sheath glow (Fig. 4a, c), these results imply that the ignition of the sheath glow comes from the tip glow. Additionally, the emission intensity shows an isotropic and exponential decay from the AIP surface, as seen in Fig. 7b. Provided that the sheath glow results from electron impact collisions with background gases, this result indicates that electrons would be ejected from the entire AIP surface.

As shown in Fig. 7c, the sheath glow peak intensity gradually increases prior to $I_{\textrm{AIP}}$ initiation, similar to tip glow, after which it rises steeply then quickly drops while the emission pattern is maintained (see Fig. 7b from $-0.39~\mu$s to 0.58 $\mu$s). This means that the sheath glow mechanism does not change during its evolution, but is enhanced while $I_{\textrm{AIP}}$ increases since the tip glow mechanism remains unchanged. From Fig. 7a, the lifetime of the sheath glow can be speculated as several microseconds.

Based on the observation results, speculations for the formation mechanism of the sheath glow are as follows. First, ectons released by ion-assisted field emission are accelerated towards the anodizing surface by as much as several electronvolts, since its surface potential is comparable with the background plasma potential. Recombination of incident ectons with surface-charged ions neutralizes the surface potential. Simultaneously, secondary electrons are ejected from the anodizing surface, leaving the surface positively charged equivalent to the ejection, called image charge formation. These secondary electrons are accelerated towards either their starting point or another ion-charging surface, strike the surface, eject other secondary electrons, and then combine with the charging ions. Although the starting point has a larger potential gradient than the ion-charge surface, secondary electron emission is suppressed by image charges that play a role in increasing the escape potential barrier. Electron multiplication occurs as the secondary electron emission yield exceeds unity^42^ for energetic incident electrons having more than several hundred electronvolts while propagating toward opposite sites from tip glow. During the multiplication, secondary electrons collide with background argon atoms and sheath glow is released. Such electron multiplication on a dielectric surface, here the anodizing film, has been reported. Hoder et al.^43^ have proved the formation mechanism of propagating streamers on a dielectric surface with the surface charge deposition concept at atmospheric pressure. Streamer electrons are deposited on a dielectric surface, and positive image charges are induced at other non-deposited surface areas. This unbalanced charge distribution forms an electric field that moves the streamer electrons towards the positively charged surface (non-deposited surface), thereby creating streamer propagation on the dielectric surface. Provided that the surface propagation mechanism applies to the sheath glow in the present work, this propagation is not measurable with the current ultra-high-speed camera as the time scale of surface propagation is several tens of nanoseconds^43^, compared to the sheath glow being recorded as accumulated images a few hundred times longer than the surface propagation.

Additionally, there is a bright and line-shape emission on the cone-shape surface that is connected to the tip glow, as marked with white arrows in the insets of Fig. 7a, from $-0.53~\mu$s to $0.58~\mu$s. It is noted that during the sheath glow period, the line-shape emission is measured in all recorded images, especially in the front-view measurements, and the sheath glow is slightly expanded in the direction of the line-shape emission. These characteristics are also well observed in the side-view measurement images in Fig. S2 (Supplementary Information). Moreover, the line-shape emission spreads from the tip glow toward the AIP outline, and the opposite side of the line-shape emission is darker. These results support that from the tip glow, electrons begin to spread out towards the AIP outline and fill the whole AIP sheath. These features also provide evidence of the secondary electron emission multiplication mentioned above.

#### Bulk glow

After termination of the sheath glow, bulk glow emerges as shown in Fig. 7b, meaning that it is a slowly emerging secondary phenomenon in the arcing evolution process. As the bulk glow is not a dominant mechanism in the early phase dynamics, discussion here is briefly provided. A detailed discussion on the whole arcing evolution process including bulk glow is forthcoming in a future work.

As shown in Figs. 5e, f, h and 7a, a short time after arcing current initiation ($t_{\textrm{avg}}$ > 0.37 $\mu$s), the sheath glow intensity abruptly decreases and bulk glow emerges. Here, the transition of the emission pattern into bulk glow is highly inhomogeneous. Its maximum intensity is not on the AIP surface but in space with a thicker region, while the bulk glow shows a clearer boundary than that of the sheath glow; for instance, at $t_{\textrm{avg}}$ = 0.37 $\mu$s, there is an ambiguous boundary (radially spreading emission), but after $t_{\textrm{avg}}$ = 1.80 $\mu$s, a clear boundary emerges as shown in Fig. 7a. The bulk glow is sustained for a few tens of microseconds, shorter than the tip glow but longer than the sheath glow.

## Conclusion

This paper investigated light emission of arcing generated on the AIP tip edge under a LTP environment with an ultra-high-speed camera. The AIP is an anodized aluminum rod with a cone-shaped head. The anodizing film at the tip edge is partially stripped to reveal the aluminum surface and localize arcing. We found light emission generated as early as 0.56 $\mu$s before arcing current initiation regarded as a usual arcing development signal. Results revealed that the tip glow from the AIP tip edge and the sheath glow from the sheath covering the AIP constitute the prior light. We proved the measurement validity of this prior light and discussed speculations for the formation mechanisms of these emissions based on the multiple snapshot image analysis. Ion-assisted field emission from the asperity on the AIP tip surface produces ectons and resultant local Ohmic heating enough to evaporate the emitting area. Those ectons collide with either evaporated aluminum atoms or argon atoms and produce both electron and photon avalanche, which is the origin of the tip glow. Those ectons also strike the anodizing film surface charged by ions from the LTP and induce secondary electron emission. Emitted secondary electrons produce electron and photon avalanches in the sheath. The electron avalanche propagates along the anodizing film due to the ion-charged anodizing surface, followed by consecutive electron and photon avalanches filling the whole sheath region, which is the origin of the sheath glow.

## Methods

### Triggering arcing events

To trigger an arcing moment, a high-voltage probe (P6139A, Tektronix Inc.) connected to the external trigger port of a digital delay generator (DG645, Stanford Research Systems) measures the voltage of the AIP ($V_{\textrm{AIP}}$). The trigger mode is set to uprising mode and the threshold level is set to 0.03 V for sensitive arcing triggering. Upon an arcing event, $V_{\textrm{AIP}}$ drastically changes, and the trigger signal is immediately transmitted to both measurement devices while data recording is paused (for instance, see Fig. 1b).

## Supplementary Information

Supplementary Information.

## Data Availability

The datasets used and/or analysed during the current study available from the corresponding author on reasonable request.

## References

[1] Zhou S (2019). Direct observation of vacuum arc evolution with nanosecond resolution. Sci. Rep..

[2] Li S (2019). Synergic effect of adsorbed gas and charging on surface flashover. Sci. Rep..

[3] Zhou S (2020). Spectroscopic study of vacuum arc plasma expansion. J. Phys. D Appl. Phys..

[4] Orr K, Yang X, Gulko I, Adamovich IV (2020). Formation and propagation of ionization waves during ns pulse breakdown in plane-to-plane geometry. Plasma Sources Sci. Technol..

[5] Michler T, Toedter O, Koch T (2020). Measurement of temporal and spatial resolved rotational temperature in ignition sparks at atmospheric pressure. Automot. Engine Technol..

[6] Slade, P. G. & Taylor, E. D. Electrical breakdown in atmospheric air between closely spaced (0.2 $$\mu$$m–40 $$\mu$$m) electrical contacts. *IEEE Trans. Compon. Packag. Technol.* **25**, 390–396, 10.1109/TCAPT.2002.804615 (2002).

[7] Li C (2021). Charge cluster triggers unpredictable insulation surface flashover in pressurized sf$$_{6}$$. J. Phys. D Appl. Phys..

[8] Saressalo A (2020). Classification of vacuum arc breakdowns in a pulsed dc system. Phys. Rev. Accel. Beams.

[9] Kasashima Y (2013). Detection of micro-arc discharge using esc wafer stage with built-in ae sensor. IEEE Trans. Semicond. Manuf..

[10] Yan E (2009). Solving arcing issues in CVD processes. ECS Trans..

[11] Yang Z (2016). Pattern dependent plasma charging effect in high aspect ratio 3D NAND architecture. IEEE ASDF.

[12] Carter D, Walde H, Nauman K (2012). Managing arcs in large area sputtering applications. Thin Solid Films.

[13] Norem J, Insepov Z, Hassanein A (2021). An integrated approach to understanding RF vacuum arcs. Sci. Rep..

[14] Timko H (2015). From field emission to vacuum arc ignition: a new tool for simulating copper vacuum arcs. Contrib. Plasma Phys..

[15] Yang W, Sun Q, Zhou Q (2020). Particle modeling of vacuum arc discharges. J. Appl. Phys..

[16] Semnani A, Venkattraman A, Alexeenko AA, Peroulis D (2013). Pre-breakdown evaluation of gas discharge mechanisms in microgaps. Appl. Phys. Lett..

[17] Anders A (2006). Physics of arcing, and implications to sputter deposition. Thin Solid Films.

[18] Kwok DTK, Yin Y, Bilek MMM, McKenzie D (2005). Enhancement of microarcing at a grounded chamber wall by nonvanishing ion sheath in a radio-frequency capacitive discharged plasma. Appl. Phys. Lett..

[19] Ling J, Boswell RW, Lafleur T, Charles C (2011). Microarcing in a helicon plasma reactor. IEEE Trans. Plasma Sci..

[20] Kim YH, Lee HS, Chang HY (2015). Micro-arc ignition on the oily surface of capacitively-coupled plasma. Curr. Appl. Phys..

[21] Norem, J. *et al.* Dark current, breakdown, and magnetic field effects in a multicell, 805 mhz cavity. *Phys. Rev. ST Accel. Beams* **6**, 072001. 10.1103/PhysRevSTAB.6.072001 (2003).

[22] Yin Y, Bilek MMM, McKenzie DR, Boswell RW, Charles C (2004). Micro-arcing in radio frequency plasmas. J. Phys. D Appl. Phys..

[23] Yin Y, Allan SY, Bilek MMM, McKenzie DR (2007). Time dependent plasma properties during microarcing in radio frequency plasmas. Appl. Phys. Lett..

[24] Yin Y, McKenzie DR, Bilek MMM (2006). Analytic analysis on asymmetrical microarcing in high plasma potential RF plasma systems. Plasma Sources Sci. Technol..

[25] Lieberman, M. A. & Lichtenberg, A. J. *Principles of Plasma Discharges and Materials Processing*, chap. 6 (Wiley &Sons. Inc., 2005), 2nd edn.

[26] Pyun S (2010). On monitoring of gas leak in the plasma vacuum process with optical emission spectroscopy. Thin Solid Films.

[27] Kasashima, Y. *et al.* In-situ detection method for wafer movement and micro-arc discharge around a wafer in plasma etching process using electrostatic chuck wafer stage with built-in acoustic emission sensor. *Jpn. J. Appl.Phys.* **53**, 03DC04. 10.7567/JJAP.53.03DC04 (2014).

[28] Oh K, Kalanov D, Anders A (2021). High-resolution observation of cathode spots in a magnetically steered vacuum arc plasma source. Plasma Sources Sci. Technol..

[29] Tektronix. P5100a 500 mhz 100x high voltage probe instructions. *Instruction manual* https://www.tek.com/ko/high-voltage-probe-manual/p5100a (2013).

[30] Gedeon V (2015). $$b$$-spline $$r$$-matrix-with-pseudostates calculations for electron collisions with aluminum. Phys. Rev. A.

[31] Lieberman, M. A. & Lichtenberg, A. J. *Principles of Plasma Discharges and Materials Processing*, chap. 3 (Wiley &Sons. Inc., 2005), 2nd edn.

[32] Engelberg EZ, Ashkenazy Y, Assaf M (2018). Stochastic model of breakdown nucleation under intense electric fields. Phys. Rev. Lett..

[33] Fowler, R. H. & Nordheim, L. Electron emission in intense electric fields. *Proc. R. Soc. Lond. Ser. A Contain. Pap. Math. Phys. Char.* **119**, 173–181. 10.1098/rspa.1928.0091 (1928).

[34] Mitchell, E. W. J. & Mitchell, J. W. The work functions of copper, silver and aluminium. *Proc. R. Soc. Lond. Ser. A. Math. Phys. Sci.* **210**, 70–84. 10.1098/rspa.1951.0231 (1951).

[35] Spataru C, Teillet-Billy D, Gauyacq J, Teste P, Chabrerie J (1997). Ion-assisted electron emission from a cathode in an electric arc. J. Phys. D Appl. Phys..

[36] Huang W-D (2022). Particle-in-cell simulation of vacuum arc breakdown process of tip-to-plate electrode configuration. J. Appl. Phys..

[37] Lide DR (1992). Ionization potentials of atoms and atomic ions. Handb. Chem. Phys..

[38] Young RD (1959). Theoretical total-energy distribution of field-emitted electrons. Phys. Rev..

[39] Yang W, Meng X, Zhou Q, Dong Z (2019). Boltzmann equation studies on electron swarm parameters in townsend breakdown of copper vapor plasma using independently assessed electron-collision cross sections. AIP Adv..

[40] Loch S, Ballance C, Wu D, Abdel-Naby SA, Pindzola M (2012). Electron-impact ionization of al. J. Phys. B: At. Mol. Opt. Phys..

[41] Bartlett PL, Stelbovics AT (2002). Calculation of total-ionization cross sections. Phys. Rev. A.

[42] Guo, J. *et al.* Secondary electron emission characteristics of al2o3 coatings prepared by atomic layer deposition. *AIP advances* **9**, 095303-1–095303-7, 10.1063/1.5113671 (2019).

[43] Jansky J, Bessieres D, Brandenburg R, Paillol J, Hoder T (2021). Electric field development in positive and negative streamers on dielectric surface. Plasma Sources Sci. Technol..

