# Supplementary material for: Observation of prior light emission before arcing development in a low-temperature plasma with multiple snapshot analysis
Source: Sci Rep. 2022 Dec 5;12:20976. doi: 10.1038/s41598-022-25550-2 (PMC9722919; doi:10.1038/s41598-022-25550-2)
Supplement: Supplementary file 1 — Supplementary Information. [file 41598_2022_25550_MOESM1_ESM.pdf]

Supplementary Information

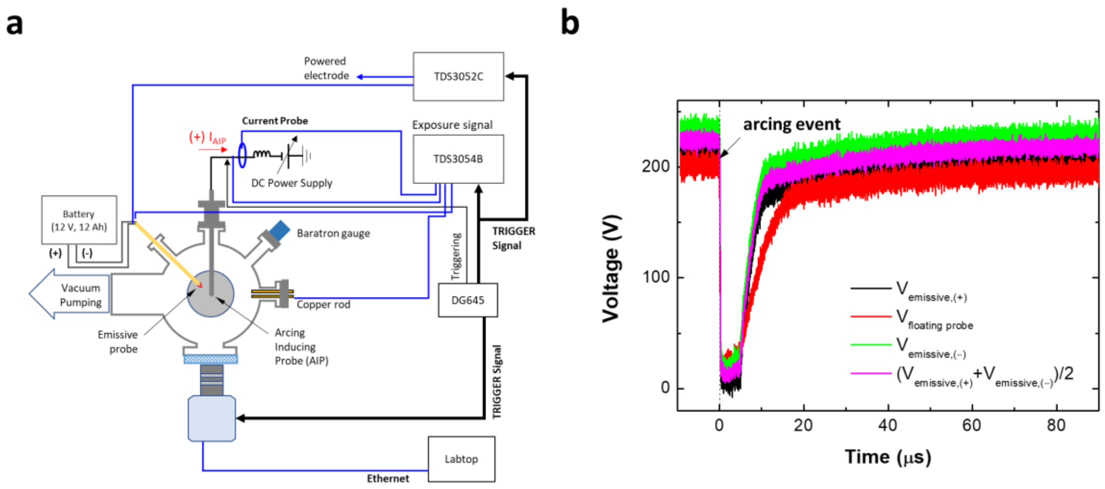

Figure S1. **(a)** Schematic diagram of the experimental setup for the measurement of plasma potential. **(b)** Voltage waveforms of the floating probe ($V_{floating\ probe}$) and positive ($V_{emissive,(+)}$) and negative terminals ($V_{emissive,(-)}$) for the emissive probe at an arcing inducing probe (AIP) voltage of −10 V, pressure of 167.9 mTorr, argon injection of 50 sccm, and RF power of 40 W. The plasma potential is taken as the average potential of the terminals (($V_{emissive,(+)}$+ $V_{emissive,(+)}$)/2).

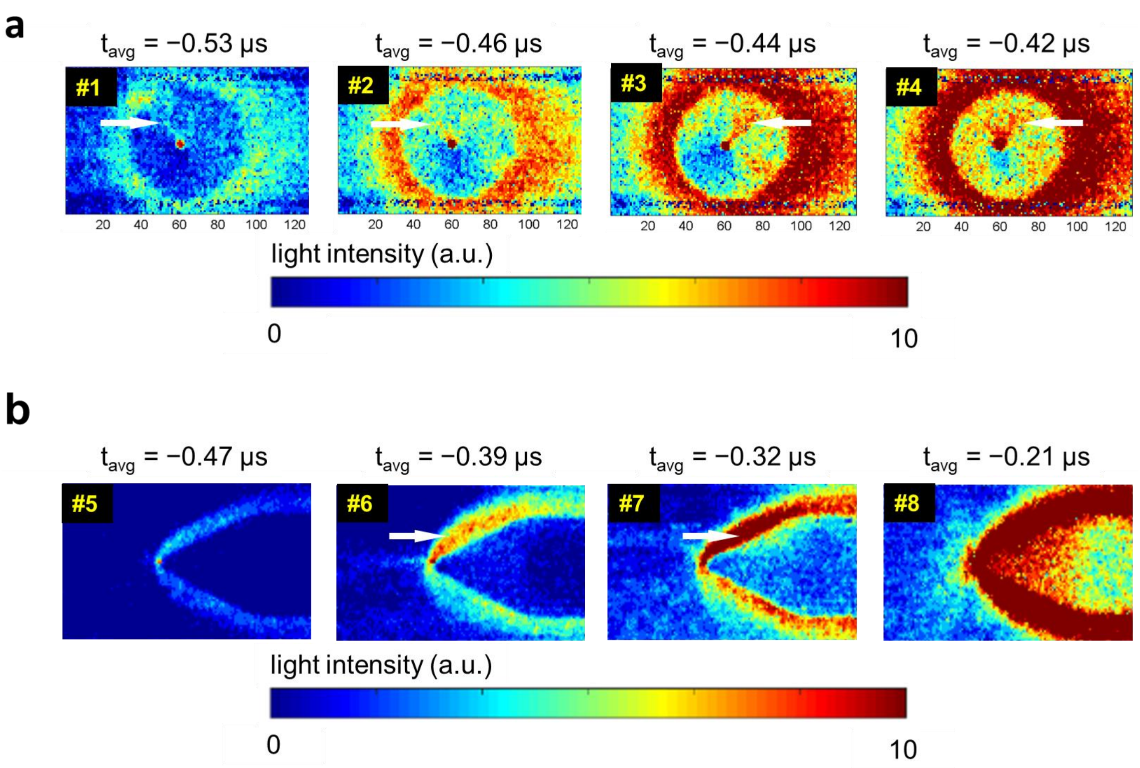

Figure S2. Recorded arcing images from **(a)** front-view and **(b)** side-view measurements at an AIP voltage of −75 V, pressure of 163.2 mTorr, argon injection of 50 sccm, and RF power of 40 W.

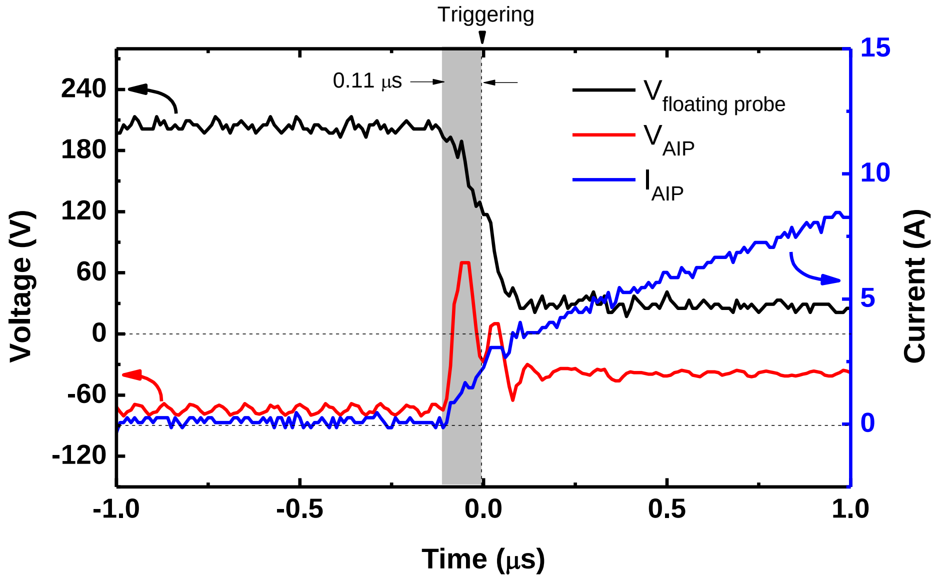

Figure S3. Voltages and current waveforms at an AIP voltage of −75 V, pressure of 167.9 mTorr, argon injection of 50 sccm, and RF power of 40 W. The gray bar indicates the time difference between the triggering moment and initiation of the AIP current.

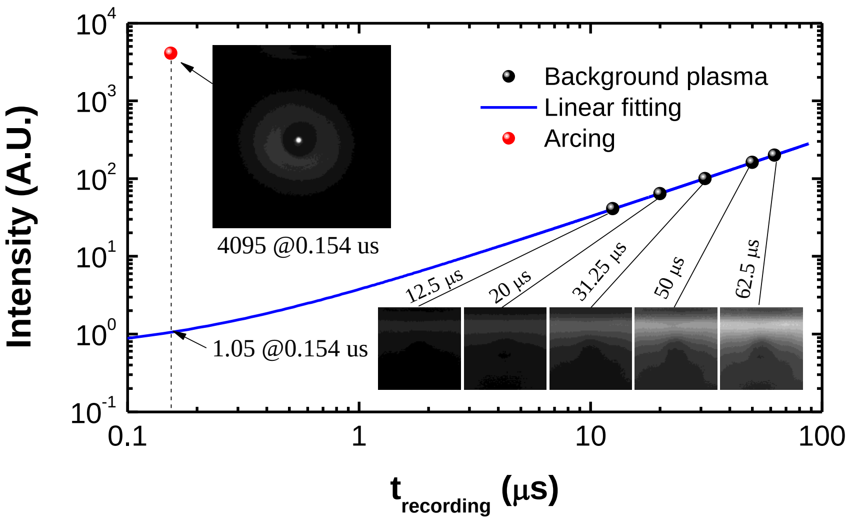

Figure S4. Emission intensity of the background plasma and arcing over recording time at an AIP voltage of −75 V, pressure of 163.2 mTorr, argon injection of 50 sccm, and RF power of 40 W.
